# Lutetium-177-PSMA I&T (Lu-PSMA) with radiosensitising capecitabine in patients with metastatic castration-resistant prostate cancer (mCRPC): results of phase Ia study

**DOI:** 10.1007/s00259-026-08006-x

**Published:** 2026-06-19

**Authors:** Claudia Leslie, Zeyad Al-Ogaili, Leone Oh, Tim Slattery, Sachin Shaji, Gabriela Marsavela, Alana Mills, Caroline Stone, Tom Ferguson

**Affiliations:** 1 Department of Medical Oncology, Fiona Stanley Hospital, Murdoch, Australia Western Australia; 2 Department of Medical Oncology, Sir Charles Gairdner Hospital, Nedlands, Australia Western Australia; 3 Department of Nuclear Medicine, Fiona Stanley Hospital, Western Australia Murdoch, Australia; 4 Medical School, University of Western Australia, Nedlands, Australia Western Australia

**Keywords:** Prostate cancer, Capecitabine, PSMA-targeted therapy, Lutetium-177, Radioligand therapy

## Abstract

**Purpose:**

Lu-PSMA is an effective therapy for mCRPC. Capecitabine is an established radiosensitiser in several solid tumours and may enhance therapeutic response when combined with radioligand therapy. We evaluated the safety and maximum tolerated dose (MTD) of capecitabine with Lu-PSMA in a heavily pre-treated mCRPC cohort.

**Methods:**

Men with mCRPC previously treated with ≥ 1 taxane and ≥ 1 androgen receptor pathway inhibitor, and deemed suitable for Lu-PSMA on PSMA-PET, were enrolled in a 3 + 3 dose-escalation study. Capecitabine was administered at four dose levels (275 mg/m^2^ – 1000 mg/m^2^) on days 1–14, with Lu-PSMA on day 10 of a 42-day cycle, for up to six cycles. The primary endpoint was the MTD; secondary endpoints included safety, PSA-50 and objective response rates (RR), PSA-progression free survival (PFS), clinical and radiological PFS, and overall survival (OS). PSA was assessed every two weeks; imaging every 12 weeks.

**Results:**

12 patients received at least one dose of Lu-PSMA. Median age was 75. No dose-limiting toxicities occurred, establishing the MTD at 1000 mg/m^2^. Common treatment-related adverse events were nausea, fatigue, and diarrhoea. Across dose levels, the PSA-50 RR was 50%, and PSA-PFS was 4.7 months (95% CI 2.3–21.6). One patient with measurable disease achieved a partial response. Exploratory PSMA PET biomarker analyses were negative, likely limited by small sample size.

**Conclusion:**

Capecitabine 1000 mg/m^2^ in combination with Lu-PSMA is safe and tolerable in heavily pre‑treated mCRPC. A dose‑expansion cohort is ongoing to further evaluate efficacy.

**Supplementary Information:**

The online version contains supplementary material available at https://doi.org/10.1007/s00259-026-08006-x.

## Introduction

Prostate cancer is the most diagnosed cancer among Australian men and the fourth leading cause of cancer-related death in 2024 [1]. Despite significant advances in systemic therapies for advanced disease [2–8], progression to metastatic castration-resistant prostate cancer (mCRPC) almost always occurs. Theranostic agents such as radionuclide therapy have shown promise in various advanced malignancies. 177Lu-PSMA therapy selectively delivers β particle radiation to prostate specific membrane antigen (PSMA)-expressing cells and is effective for patients with PSMA-avid mCRPC [9, 10]. The VISION study demonstrated a survival benefit with 177Lu-PSMA therapy in patients with PSMA-positive mCRPC who had previously received at least one androgen receptor pathway inhibitor, and one or two taxane-based chemotherapies or were taxane-ineligible [11].

Theranostic agents have been used successfully in other malignancies [12–14], both alone and in combination with systemic therapies. The combination of 177Lu-DOTATATE with radiosensitising oral chemotherapy capecitabine has shown promising efficacy in the treatment of metastatic neuroendocrine tumours without evidence of prohibitive or unexpected additional toxicity [15–17]. Capecitabine is an oral prodrug of 5-fluorouracil which undergoes final activation via thymidine phosphorylase, an enzyme expressed at significantly higher levels in prostate cancer tissue compared with normal prostate tissue, enhancing its therapeutic index and supporting its use as a radiosensitiser. Clinically, capecitabine has demonstrated modest activity as a single agent in prostate cancer [18], and whilst various pharmacotherapies have been investigated in combination with Lu-PSMA treatment [19–25], no study has evaluated the addition of capecitabine as a radiosensitiser. Here, we report the dose escalation phase of the LuCape trial, which evaluated the optimal dose and safety of adding radiosensitising capecitabine to Lu-PSMA in heavily pretreated patients with mCRPC. Recruitment to the dose‑expansion cohort is ongoing.

## Materials and methods

### Patient population

Eligible participants were men aged ≥ 18 years with a histopathological or clinical diagnosis of mCRPC who had experienced disease progression with a rising prostate specific antigen (PSA) defined by PCWG3 criteria [26] after at least one taxane chemotherapy for mCRPC and one androgen receptor pathway inhibitor (ARPI) for mCRPC or castration-sensitive disease. Participants with a known BRCA mutation were required to have received a poly (ADP-ribose) polymerase inhibitor. Participants were required to have imaging evidence of metastatic disease on whole-body bone scan or computerised tomography (CT) scan, and have an Eastern Cooperative Oncology Group (ECOG) performance status of 0–2 with adequate organ function. Participants were required to have PSMA-avid disease without fluorodeoxyglucose (FDG) discordance on dual positron emission tomography (PET) scans. All patients underwent dihydropyrimidine dehydrogenase (DPD) genotyping or provided historical evidence of testing; patients with DPD enzyme deficiency were excluded. Full eligibility criteria are provided in the study protocol.

### Trial design and interventions

This was a prospective, investigator-initiated, single-arm, single-centre phase 1a/1b dose-escalation and dose-expansion study. A 3 + 3 dose escalation design was employed, using a modified Fibonacci schema with four fixed dose levels of oral capecitabine based on body-surface area; 275 mg/m^2^, 550 mg/m^2^, 825 mg/m^2^, 1000 mg/m^2^. Capecitabine was provided as 500 mg and 150 mg film-coated tablets and given twice daily on days 1–14 of a 42-day cycle. Lu-PSMA was administered intravenously on day 10 of each cycle at a dose of 8.0 GBq (± 10%), with SPECT/CT conducted on day 11. This schedule was chosen to balance effective radiosensitisation with tolerability. Treatment was continued for up to six cycles unless discontinued earlier due to disease progression, prohibitive toxicity, or loss of PSMA avidity, defined as markedly reduced uptake on day 11 SPECT/CT suggesting minimal ongoing benefit. Participants could continue therapy beyond progression if the investigator deemed there was ongoing clinical benefit.

Dose escalation rules are detailed in Figure S1. The maximum tolerated dose (MTD) was the highest dose level achieved with < 33% of participants experiencing a dose-limiting toxicity (DLT) during the first 42-day cycle. Criteria for DLT are listed in the supplementary material. Patients must have received at least 75% of planned doses to be considered evaluable.

### Imaging procedures and analysis

All participants underwent PSMA PET/CT and ^18^F-FDG PET/CT imaging as part of screening and eligibility assessment. PSMA PET/CT was performed using either ^68^Ga-labelled PSMA or ^18^F-PSMA-DCFPyL, according to tracer availability and institutional practice. Prior PET/CT studies performed within 60 days of consent were permitted. PET/CT acquisition and reconstruction protocols were harmonised across scanners using phantom-based quality assurance to ensure comparability of quantitative measures. Eligibility required a maximum standardised uptake value (SUVmax) > 15 in at least one lesion and > 10 at all sites of measurable disease. FDG discordance was defined as the presence of FDG-avid lesions without corresponding PSMA-avid disease, suggesting tumour biology unlikely to benefit from PSMA-targeted therapy.

Lutetium-177–labelled PSMA therapy was administered using the PSMA I&T ligand (^177^Lu-PSMA-I&T) in accordance with institutional procedures. ^177^Lu-PSMA-I&T was used due to institutional availability at the time the trial was conducted and has shown comparable efficacy and toxicity profile to ^177^Lu-PSMA-617 [27]. ^177^Lu-PSMA-I&T was delivered at 8.0 GBq (+/-10%) per cycle on day 10 of each 42-day treatment cycle. Radiopharmaceutical preparation, quality control, and release were performed in compliance with local radiopharmacy and regulatory standards. Treatment was administered via peripheral intravenous infusion followed by a saline flush. Patients received supportive care at the investigator’s discretion. Post-therapy whole-body scintigraphy and SPECT/CT of the chest, abdomen, and pelvis were performed approximately 24 hours after each administration to assess biodistribution and tumour uptake.

Image reconstruction and processing were performed using Syngo.via / Symbia.net software (Siemens Healthineers), in accordance with institutional protocols. Post-therapy imaging was reviewed qualitatively to assess radiotracer distribution and relative tumour uptake across cycles. Loss of PSMA avidity was defined as a marked qualitative reduction in tumour uptake compared with prior cycles, suggesting limited ongoing benefit.

Exploratory quantitative imaging analyses were performed on baseline PSMA PET/CT scans. Tumour segmentation was conducted using MIM Software (MIM Software Inc.) with whole-body semi-automated segmentation applying a fixed SUV threshold of 3.0. Quantitative metrics extracted included SUVmax, whole-body tumour SUVmean, PSMA tumour volume, and PSMA Total (defined as the product of whole-body PSMA tumour volume and whole-body tumour SUVmean). These metrics were analysed for association with treatment response and clinical outcomes. Given the small sample size, all imaging analyses were considered exploratory and hypothesis-generating.

### Trial oversight

The study protocol was approved by the South Metropolitan Health Service Human Research Ethics Committee (RGS0000005919), registered with the Australian New Zealand Clinical Trials Registry (ACTRN12623001072606), and supported by South Metropolitan Health Service, a division of the Western Australian Department of Health, with additional grants received from The Hospital Research Foundation Group (formerly Spinnaker Health Research Foundation). The study was conducted in accordance with the Declaration of Helsinki, Good Clinical Practice (GCP) guidelines, and all relevant regulatory requirements. All participants provided written informed consent prior to enrolment.

### Endpoints and assessments

The primary endpoint was the MTD and recommended phase Ib dose of capecitabine administered concurrently with Lu-PSMA.

The key secondary endpoint was safety, defined by the frequency, timing, and severity of grade ≥ 3 adverse events, assessed according to Common Terminology Criteria for Adverse Events (CTCAE) version 5.0. Additional secondary endpoints included PSA response rate (≥ 50% reduction from baseline), objective response rate (ORR) using RECIST 1.1 for soft tissue lesions [28] and PCWG3 criteria for bone lesions [26]; radiological progression-free survival (rPFS), defined as the time from registration to radiographic progression or death; PSA-PFS per PCWG3 criteria [26]; clinical PFS (cPFS), defined as the time from registration to the earliest occurrence of radiographic progression, symptomatic progression, initiation of new systemic therapy, or death; and overall survival (OS), defined as the time from registration to death from any cause. Baseline PSA was defined as the last PSA measurement obtained prior to cycle 1.

Adverse events were assessed starting with the first dose and continuing until 12 weeks after the final treatment. Clinical and laboratory assessments were performed on days 1, 15 and 29 of each cycle, and then every 12 weeks until disease progression. Imaging, including CT of the chest, abdomen, and pelvis, and whole-body bone scan, were performed every 12 weeks until progression.

### Statistical analysis

All data were captured prospectively in a REDCap database and subsequently exported for analysis. Data cleaning and statistical analyses were performed using Python (version 3.12.7) within a controlled analysis environment. Descriptive statistics were calculated for baseline demographics and safety data. Treatment efficacy was assessed by calculating percentage change in PSA from baseline pre-treatment value to the nadir PSA. PFS and OS were estimated via Kaplan-Meier method with 95% confidence intervals. Data visualisation was undertaken using both Python (Seaborn and Matplotlib) and Microsoft Power BI. Exploratory analyses assessed associations between imaging-based parameters with PSA response using Wilcoxon rank‑sum testing for non-parametric data, Spearman’s correlation for continuous data, and univariate logistic regression for predictors of response.

## Results

Sixteen patients were recruited between October 2023 and May 2025. Two patients withdrew consent before starting treatment, another two did not meet screening criteria. No dose limiting toxicities were observed, recruitment for the dose-escalation phase stopped at 12 participants. Baseline characteristics of the 12 participants (intention-to-treat population) are shown in Table 1. All participants had received cabazitaxel for mCRPC. All received at least one cycle of treatment and were included in the safety analysis. As of the data cut-off (8 December 2025), the median number of cycles received was 4. At this time, two patients were continuing treatment, three had completed all 6 cycles of therapy, two patients ceased Lu-PSMA early due to perceived lack of further benefit, four discontinued due to radiological progression, and two ceased due to clinical progression. No patients discontinued treatment because of treatment-related toxicity.

**Table 1:** Participants baseline characteristics

	*N* = 12 (%)
Age (median)	74.5 years (69–78)
ECOG	
0 1	4 (33) 8 (67)
Location of metastases^#^	
Lymph node only Bone only Visceral disease	0 7 (58) 2 (17)
Median baseline PSA	25 (13.0-54.5)
Previous ARPI	
Enzalutamide Abiraterone Other	11 (91) 1 (8) 1 (8)
Previous chemotherapy for CSPC	7 (58)
Previous chemotherapy for CRPC	
Docetaxel Cabazitaxel	5 (42) 12 (100)

Qualitative data is presented as absolute count and percentages, continuous data are presented as median and interquartile range)

#location of metastases on conventional imaging

### Safety

The MTD was 1000 mg/m^2^, with no dose-limiting toxicities identified during the dose escalation. All patients experienced adverse events (AEs), and 25% experienced grade ≥ 3 AEs. Reported treatment-related AEs are listed in Table 2. The most common treatment-related AEs were nausea and fatigue, followed by diarrhoea and dry mouth. AEs were more frequent during cycle 1 (see Figure S2).

**Table 2:** Treatment emergent and treatment-related adverse events

Adverse event	Participants with TEAE any grade (%)	Participants with TEAE grade ≥ 3 (%)	Participants with TRAE any grade (%)	Participants with TRAE grade ≥ 3 (%)
Pain	12 (100)	2 (17)	2 (17)	
Nausea	8 (67)	1 (8)	7 (58)	1 (8)
Fatigue	7 (58)		7 (58)	
Constipation	6 (50)		3 (25)	
Diarrhoea	5 (42)		5 (42)	
Infection	5 (42)	1 (8)	1 (8)	
Dry Mouth	4 (33)		4 (33)	
Anaemia	3 (25)		2 (17)	1 (8)
Anorexia	3 (25)		1 (8)	
Fall	2 (17)			
Fever	2 (17)		1 (8)	1 (8)
Haematuria	2 (17)		1 (8)	
Lower urinary tract symptoms	2 (17)			
Rash	2 (17)			
Atrial fibrillation	1 (8)			
Bloating	1 (8)			
Chest swelling	1 (8)			
Cough	1 (8)			
Dizziness	1 (8)			
Dry eyes	1 (8)		1 (8)	
Dry skin	1 (8)		1 (8)	
Dyspnea	1 (8)			
Gum Pain	1 (8)		1 (8)	
High Alkaline Phosphatase	1 (8)		1 (8)	
Hypocalcaemia	1 (8)			
Injection site reaction	1 (8)			
Localised oedema	1 (8)			
Lymphocyte Decrease	1 (8)		1 (8)	
Myalgia	1 (8)			
Neutropaenia	1 (8)		1 (8)	
Oedema limbs	1 (8)		1 (8)	
Peripheral Sensory Neuropathy	1 (8)		1 (8)	
Pneumonia	1 (8)	1 (8)		
Thrombocytopaenia	1 (8)			
Vomiting	1 (8)		1 (8)	
Weight Loss	1 (8)		1 (8)	

TEAE = treatment-emergent adverse events; TRAE = treatment related adverse events

Seven serious AEs were reported during the study period; two (nausea, fever) were deemed treatment-related. At data cut-off, five patients had died, none while on study treatment. Regarding AEs of special interest, diarrhoea occurred in five participants (all grade 1) and hand–foot syndrome in none. Potentially overlapping toxicities included anaemia in three participants (one participant with grade 3 anaemia) and thrombocytopenia in one.

### Treatment response

**Fig. 1:**
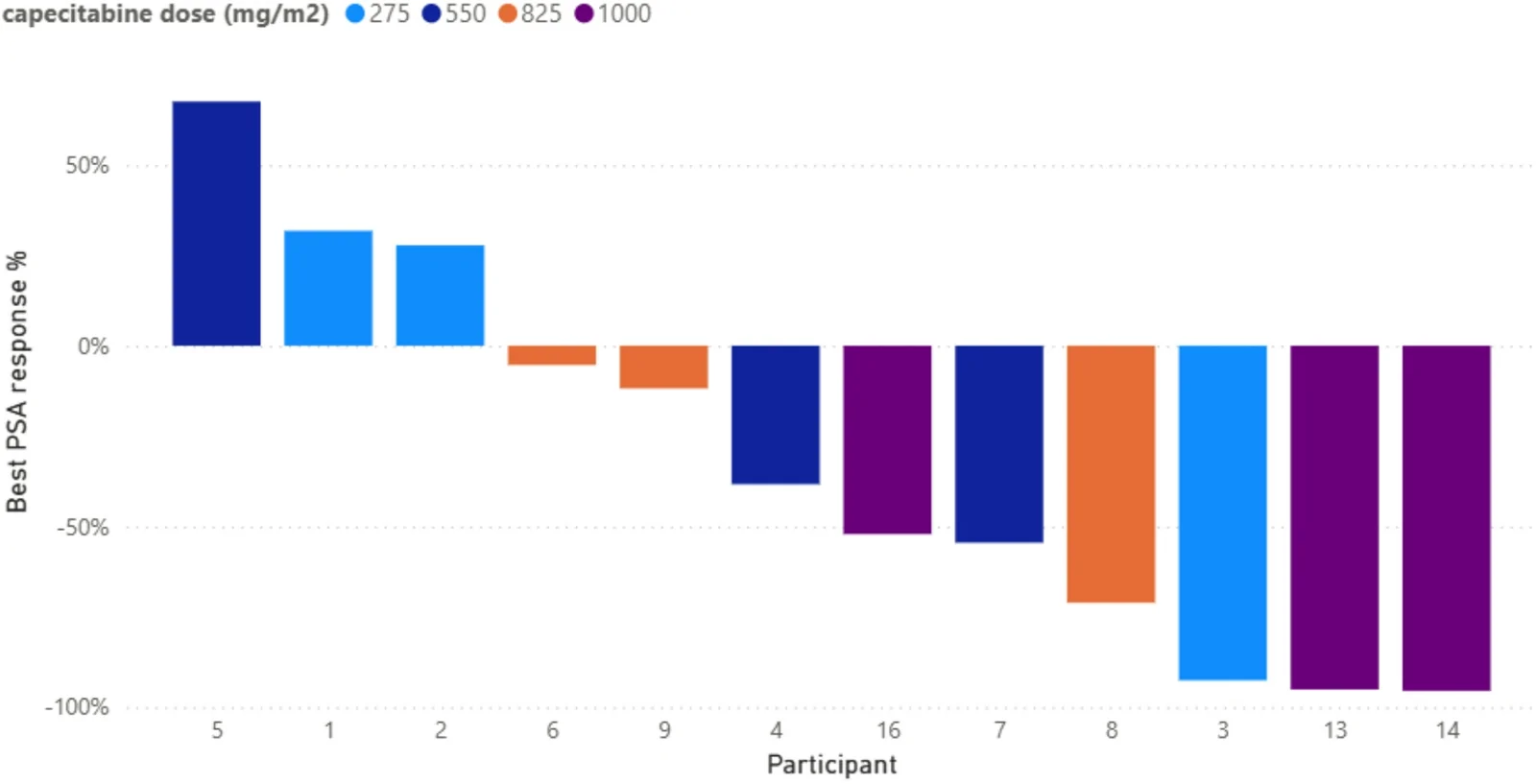
Best PSA response by dose level (% change from baseline)

**Fig. 2:**
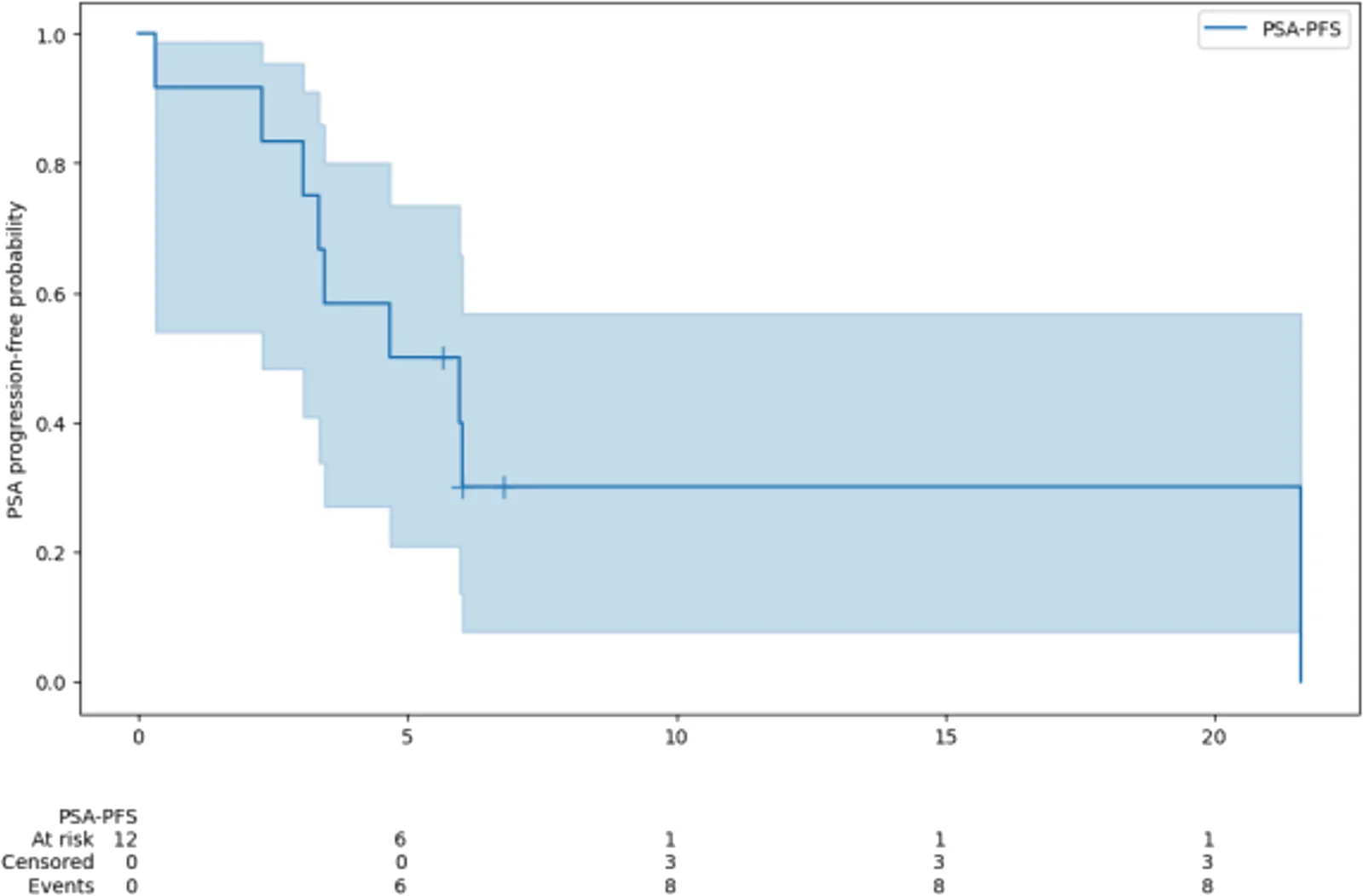
PSA progression-free survival for whole cohort

Six participants (50%) had a ≥ 50% PSA reduction from baseline, five of which were confirmed on repeat PSA. Nine participants had any PSA reduction from baseline, with a median percentage decline in these participants of 54.5% (range = 5.3–95.5) in this heavily pre‑treated cohort. Best PSA response by dose level is shown in Fig. 1. Nine patients had PSA progression at data cut‑off, with median PSA-PFS of 4.7 months (95% CI 2.3–21.6) (Fig. 2). For best radiological response, one patient achieved a partial response (ORR 8%), eight had stable disease and three had progressive disease. Median clinical PFS was 10.0 months (95% CI 2.8–12.8) (Figure S3). At data cut‑off, seven deaths had occurred, with median OS of 11.7 months (95% CI 4.8–16.1) (Figure S4) .

### Baseline PSMA PET metrics and PSA response

Baseline PSMA PET metrics were evaluable in all 12 patients. The median baseline SUVmax was 30.1 (IQR 16.9), median SUVmean was 6.85 (IQR 1.95), median total PSMA uptake 1,173 (IQR 2,016.8), and median PSMA tumour volume 204.5 mL (IQR 226.4).

**Fig. 3:**
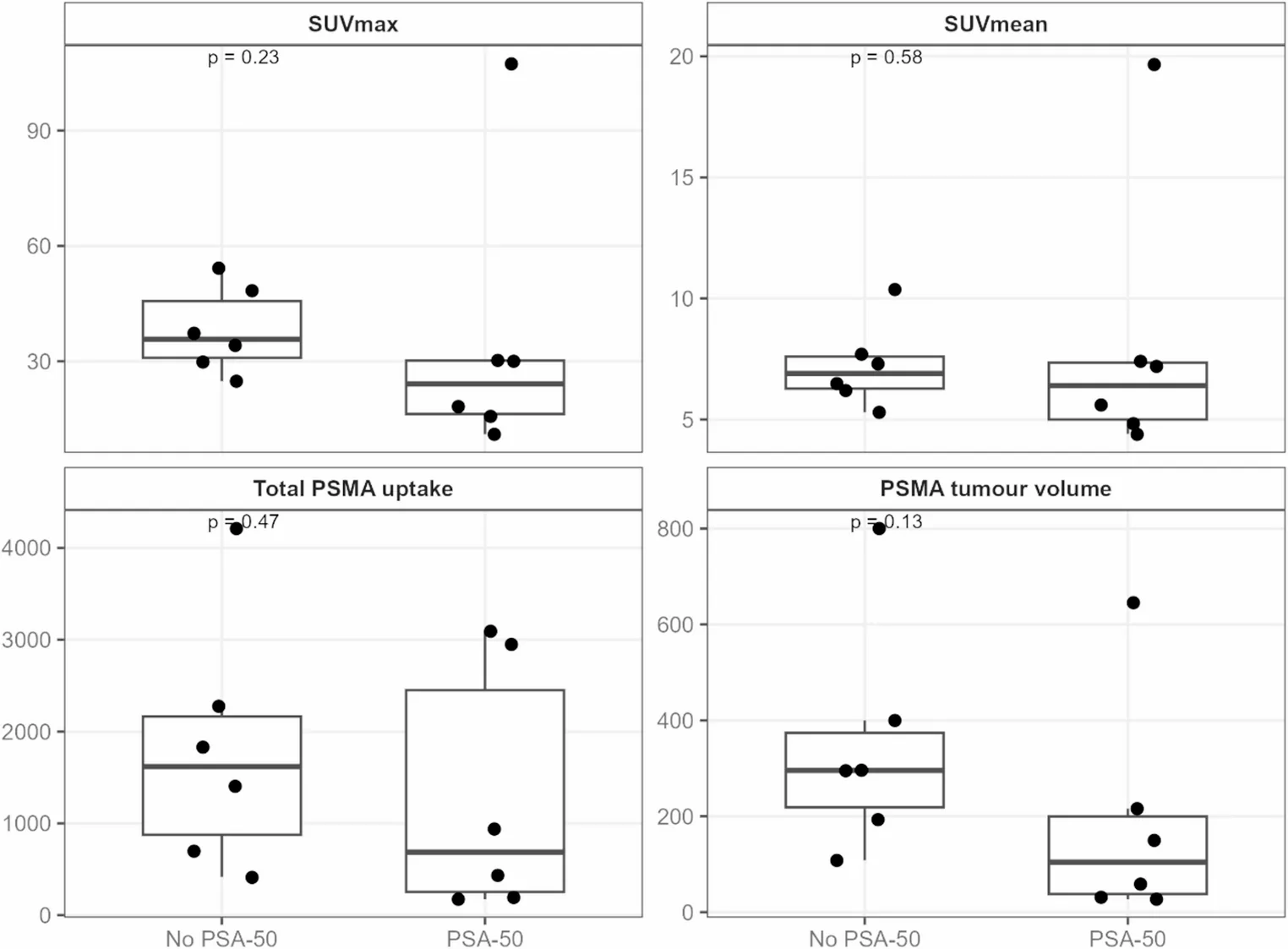
Baseline PSMA PET metrics according to PSA-50 response. Boxplots show baseline SUVmax, SUVmean, total PSMA uptake, and PSMA tumour volume stratified by PSA-50 response status. No statistically significant differences were observed between responders and non-responders

Patient-level PSMA PET metrics and PSA response are shown in Table S1. There were no statistically significant differences in baseline SUVmax, SUVmean, total PSMA uptake, or PSMA tumour volume between PSA-50 responders and non-responders (all Wilcoxon *p* > 0.1) (Fig. 3). Comparisons between responders and non-responders showed no significant association for SUVmax (*p* = 0.24), SUVmean (*p* = 0.59), total PSMA uptake (*p* = 0.48), or PSMA tumour volume (*p* = 0.13). PSA-50 responses were observed across a wide range of baseline PSMA expression levels and tumour burden (Table S1).

## Discussion

There is a growing clinical need for new therapies in metastatic prostate cancer, as earlier use of ARPIs and taxane chemotherapy leaves few effective options once treatment‑refractory mCRPC develops. In this phase Ia study, we demonstrated oral capecitabine at 1000 mg/m^2^ is safe and tolerable when combined with Lu-PSMA as a radiosensitiser in heavily pre-treated mCRPC. There were no dose-limiting toxicities, and rates of serious treatment-related adverse events were low. Additionally, few grade ≥ 3 toxicities typically associated with capecitabine or expected from dual therapy were observed. Notably, there were no instances of hand-foot syndrome, likely owing to the lower dosing and longer off-treatment interval compared with standard capecitabine regimens without accompanying radiation therapy. Diarrhoea, however, remained common, consistent with known gastrointestinal toxicity reported with Lu‑PSMA monotherapy [29] and capecitabine. Overall, adverse-event rates were similar to Lu-PSMA alone [30] and comparable to other combination Lu-PSMA regimens, with the exception of a slightly higher incidence of diarrhoea [11, 31, 32].

The dose of 1000mg/m^2^ of capecitabine twice daily is higher than that used in radiosensitisation regimens in other tumour types such as rectal and pancreatic cancer (825mg/m^2^ and 830mg/m^2^ respectively) [33, 34]. In our study capecitabine was only given for 14 days around the administration of Lu-PSMA with a 28-day off-treatment period between cycles, which was chosen to complement the standard dosing schedule of Lu-PSMA, and to maximise radiosensitisation effect whilst minimising toxicity. Combination capecitabine with external beam radiation generally requires administration of capecitabine every day of radiation, resulting in higher total medication exposure within the same time period. In line with this, rates of hand-foot syndrome were markedly lower in this study, but rates of diarrhoea was similar [33]. Rates of toxicity of this combination should be verified in a larger phase II study.

Whilst this phase I study primarily evaluated dose and safety, the PSA response rate observed is encouraging and consistent with published Lu‑PSMA studies [11, 30]. Most patients (75%) experienced a PSA decline from baseline. A previously reported phase II trial of Lu-PSMA monotherapy demonstrated PSA‑50 responses in 57% of participants, median PSA-PFS of 7.6 months and OS of 13.5 months [30]. The VISION trial, which compared Lu-PSMA-617 plus protocol permitted standard of care versus standard of care alone, demonstrated a PSA-50 response rate of 46%, and OS of 15.3 months [11]. Participants in these studies, however, generally had less prior taxane exposure, with fewer receiving ≥ 2 chemotherapy lines. Despite greater prior treatment in our cohort, PSA response rates were comparable, suggesting a potential treatment‑enhancing effect of capecitabine that warrants further investigation in a larger cohort.

A range of systemic therapies have been combined with Lu-PSMA to augment antitumour response. Investigated combinations include the ARPI enzalutamide [21] poly-ADP ribose polymerase inhibitor Olaparib [32], immune checkpoint inhibitors and a flavonoid genistein derivate indrinoxil (NOX66) [20]. Patient populations in these studies varied; for example, the Enza-P study observed higher PSA response rates, but included less heavily pre‑treated patients [21]. Toxicities have also differed; Lu-PSMA plus NOX66 was associated with increased anaemia and perianal inflammation [20], whereas Lu-PSMA with dual immune-checkpoint blockade resulted in excess myocarditis [25]. No phase III trials have yet compared these combinations with standard of care, and multiple trials exploring novel combinations remain ongoing [22].

Baseline PSMA PET metrics were explored as potential predictors of biochemical response; however, no statistically significant associations were identified between baseline SUVmax, SUVmean, total PSMA uptake, or PSMA tumour volume and PSA-50 response. This is consistent with the phase I nature of the LuCape study, which was designed for safety assessment and dose selection rather than biomarker validation, and lacked power to detect modest imaging‑response correlations.

In contrast to prior studies reporting an association between baseline SUVmean and treatment response [35, 36], no clear relationship was observed here. This likely reflects the small cohort size, the exploratory nature of the analysis, and potential confounding by combination therapy rather than the lack of biological relevance. Larger studies incorporating longitudinal imaging and dosimetry-guided assessments will be needed to clarify the prognostic and predictive value of PSMA PET metrics in combination radioligand therapy [37].

## Conclusion

This study demonstrates that capecitabine 1000 mg/m^2^ in combination with Lu-PSMA treatment is tolerable for patients with mCRPC. Further data from the dose expansion cohort are awaited to assess efficacy.

## Supplementary Information

Below is the link to the electronic supplementary material.


Supplementary Material 1 (PDF 228 KB)


## Data Availability

The datasets generated during and/or analysed during the current study are available from the corresponding author on reasonable request.

## Declarations

### Registered with the Australian New Zealand Clinical Trials Registry 6/10/2023

https://anzctr.org.au/Trial/Registration/TrialReview.aspx?id=386489&isReview=true
