# Supplementary material for: Lutetium-177-PSMA I&T (Lu-PSMA) with radiosensitising capecitabine in patients with metastatic castration-resistant prostate cancer (mCRPC): results of phase Ia study
Source: Eur J Nucl Med Mol Imaging. 2026 Jun 19;53(11):6452–60. doi: 10.1007/s00259-026-08006-x (PMC13526046; doi:10.1007/s00259-026-08006-x)
Supplement: Supplementary file 1 [file 259_2026_8006_MOESM1_ESM.pdf]

Supplementary appendix

Title: Lutetium-177-PSMA I&T (**Lu**-PSMA) with radiosensitising **Cape**citabine in patients with metastatic castration-resistant prostate cancer (mCRPC): Results of phase Ia study

Authors: Claudia Leslie[1], Zeyad Al-Ogaili[2,4], Leone Oh[1,3,4], Tim Slattery[1], Sachin Shaji[1], Gabriela Marsavela[1], Alana Mills[1], Caroline Stone[1], Tom Ferguson[1,4]

Affiliations:
1. Department of Medical Oncology, Fiona Stanley Hospital, Murdoch, WA, Australia
2. Department of Nuclear Medicine, Fiona Stanley Hospital, Murdoch, WA, Australia
3. Department of Medical Oncology, Sir Charles Gairdner Hospital, Nedlands, WA, Australia
4. Medical School, University of Western Australia, Nedlands, WA, Australia

European Journal of Nuclear Medicine and Molecular Imaging (EJNMMI)

Contact information:
Dr Claudia Leslie
Fiona Stanley Hospital, 11 Robin Warren Drive, Murdoch, Western Australia 6150.
Phone: +61 8 6152 2222, fax number: +61 8 6152 4080
E-mail address: Claudia.leslie@health.wa.gov.au

Supplementary appendix – contents

Figure S1	2
Figure S2	3
Dose limiting toxicity rules	4
Figure S3	5
Figure S4	6
Table S1	7

Figure S1. Dose escalation rules for capecitabine

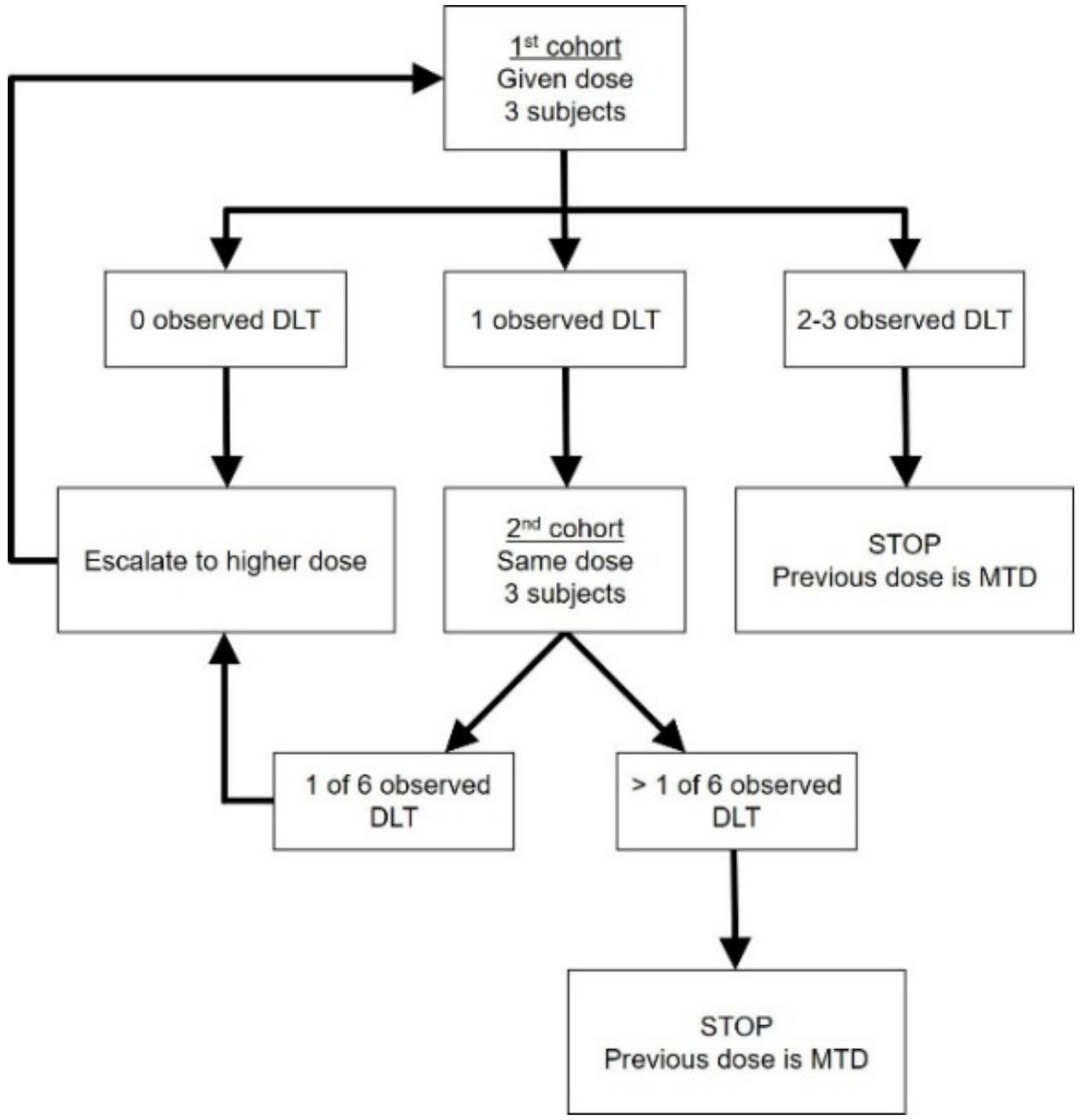

Figure S2. Timing of treatment related adverse events

Adverse Event	C1	C2	C3	C4	C5	Total
Abdominal Pain		1				**1**
Anaemia	2	2	1			**5**
Anorexia		1				**1**
Constipation	1	1	2			**4**
Diarrhoea	5	1		1		**7**
Dry Mouth	3	1				**4**
Dry Skin	1		1			**2**
Edema Limbs	1					**1**
Fatigue	5	2	1			**8**
Fever		1				**1**
Gum Pain		1				**1**
Haematuria			1			**1**
High Alkaline Phosphatase	1					**1**
Lymphocyte Decrease					1	**1**
Musculoskeletal Chest Pain	1					**1**
Nausea	3	4				**7**
Neutropaenia				1		**1**
Peripheral Sensory Neuropathy	1					**1**
Urinary Tract Infection			1			**1**
Vomiting				1		**1**
Weight Loss					1	**1**
**Total**	**24**	**15**	**7**	**3**	**2**	**51**

Dose limiting toxicity (DLT) rules

A DLT:

- Is defined as any of the TRAEs listed below, as defined by the National Cancer Institute's Common Terminology Criteria for Adverse Events, version 5.0 (CTCAE v5.0).
- Occurs during the first 42 days of treatment, provided that the participant has received at least 75% of planned doses.
- Arises from an AE that is not reasonably attributed to the patient's underlying disease, other medical condition or concomitant medications.

The DLT definitions are:

1. Grade 3 haematologic or non-haematologic toxicity, excluding:
    a. Grade 3 aspartate aminotransferase (AST), alanine aminotransferase (ALT), alkaline phosphatase (ALP), or gamma-glutamyl transferase (GGT) elevation for less than 7 days.
    b. First occurrence of Grade 3 electrolyte abnormalities resolving to less than Grade 2 within 48 hours with supportive treatment.
    c. Grade 3 fatigue, asthenia, nausea, or other manageable constitutional symptoms.
    d. Grade 3 vomiting or diarrhoea that lasts for less than 48 hours with antiemetic or antidiarrhoeal medications.
2. Grade 4 haematologic or non-haematologic toxicity, excluding:
    a. First occurrence of Grade 4 electrolyte abnormalities resolving to less than Grade 2 within 24 hours with supportive treatment.
    b. Grade 4 fatigue, asthenia, nausea, or other manageable constitutional symptoms.
    c. Grade 4 vomiting or diarrhoea that lasts for less than 24 hours with antiemetic or antidiarrhoeal medications.
3. Any toxicity, regardless of the CTCAE grade, resulting in discontinuation, dose reduction or treatment with less than 75% of planned doses, will be reviewed by the study team, and will be considered a DLT if the study team determines the toxicity cannot be attributed to the patient's underlying disease, other medical condition or concomitant medications.

Figure S3. Clinical progression-free survival for all dose levels

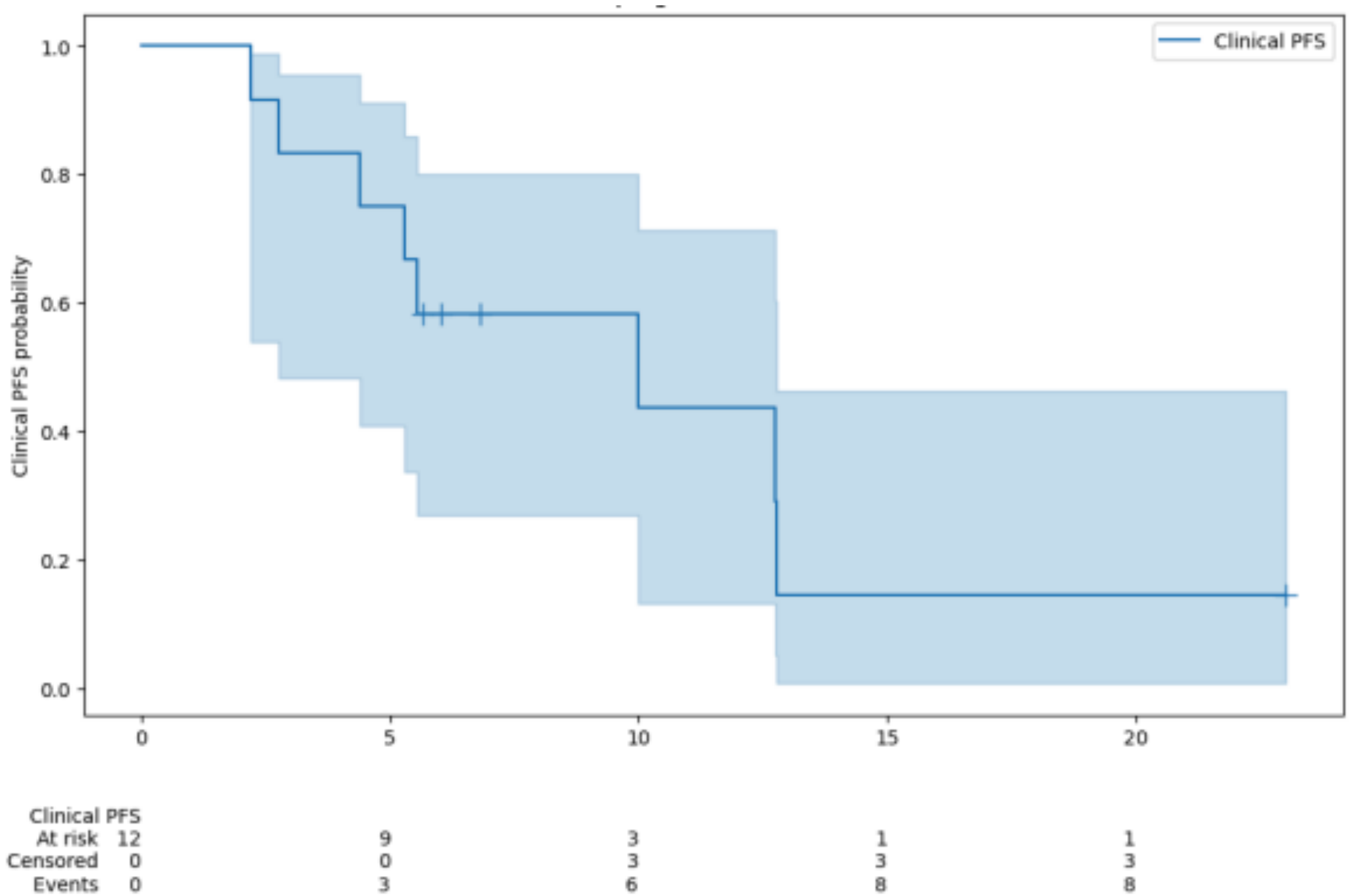

Figure S4. Overall survival for all dose levels

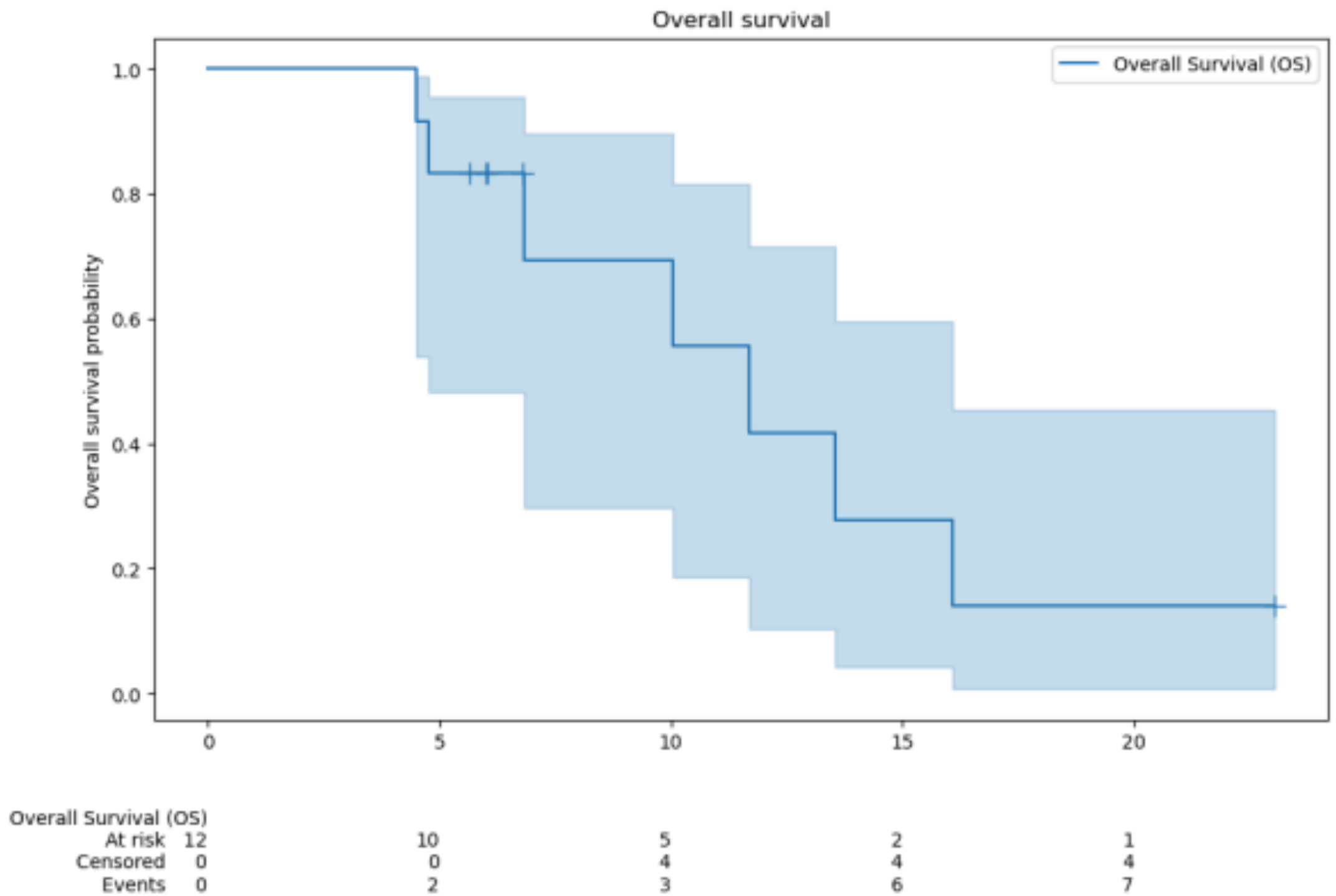

Supplementary Table S1. Patient-level baseline PSMA PET metrics and PSA response

Baseline PSA was defined as pre cycle 1 PSA measurement. PSA-50 response was defined as a ≥50% decline from baseline PSA

Participant	Baseline PSA	Best PSA % change	SUVmax	SUVmean	PSMA total	PSMA tumour volume	Sites of disease per PSMA PET
1	6.6	31.8	37.3	6.5	700	108	Bone and lymph nodes
2	13.3	27.8	24.8	5.3	4211	800	Bone only
3	65	-92.6	107.4	19.7	2955	150	Bones and lymph nodes
4	120	-38.3	48.4	10.4	417	400	Bone and lymph nodes
5	370	67.6	29.8	6.2	1831	295	Bone and liver
6	19	-5.3	54.2	7.7	2276	296	nodes and adrenal
7	22	-54.5	18.2	5.6	174	31	Bone only
8	28	-71.1	15.6	4.8	3088	645	Bone and adrenal
9	51	-11.8	34.1	7.3	1405	193	Bone and lymph nodes
13	12	-95.1	30.2	7.4	433	58.5	Bone only
14	9.8	-95.5	30	7.2	194	27	Bone only
16	48	-52.1	11	4.4	941	216	Bone only
